# Does breastfeeding account for the association between maternal sensitivity and infant cognitive development in a large, nationally representative cohort?

**DOI:** 10.1186/s12887-022-03133-4

**Published:** 2022-01-26

**Authors:** P. Nina Banerjee, Karen E. McFadden, Jacqueline D. Shannon, Leslie L. Davidson

**Affiliations:** 1 Department of Epidemiology, Columbia University Mailman School of Public Health, New York, USA; 2 Department of Early Childhood Education and Art Education, Brooklyn College, CUNY, New York, USA

**Keywords:** Maternal sensitivity, Parenting, Cognitive development, Breastfeeding, Maternal depression

## Abstract

**Background:**

Previous research has established that exposure to high maternal sensitivity is positively associated with advances in infant cognitive development. However, there are many fixed and modifiable factors that influence this association. This study investigates whether the association between maternal sensitivity and infant cognitive development in the first year of life is accounted for by other factors, such as breastfeeding, maternal depressive symptoms, maternal alcohol use, infant birth weight or demographic covariates.

**Methods:**

Using data from the Early Childhood Longitudinal Study-Birth (ECLS-B) Cohort, a nationally representative sample of U.S. born children, multi-variable regression analyses was used to examine whether breastfeeding, maternal depressive symptoms and alcohol use were associated with maternal sensitivity, as measured by the Nursing Child Assessment Teaching Scale (NCATS), and with infant cognitive development, as measured by the Bayley Scales of Infant Development, Short Form, Research Edition, after controlling for demographic covariates (infant sex, maternal age, education, race/ethnicity, income, parity, family structure) and infant birth weight.

**Results:**

Breastfeeding, depressive symptoms and alcohol use were not associated with maternal sensitivity scores after controlling for demographic covariates and infant birth weight. However, breastfeeding (β = .079, *p* < .001), depressive symptoms (β = −.035, *p* < .05), and maternal sensitivity (β = .175, *p* < .001) were each significantly associated with infant cognitive development scores, even after controlling for demographic covariates and birthweight (R^2^ = .053, *p* < .001). The association between maternal sensitivity and infant cognitive development did not attenuate after adjusting for breastfeeding. Instead, both sensitivity and breastfeeding independently contributed to higher infant cognitive development scores.

**Conclusion:**

Maternal sensitivity and breastfeeding are separate means to advancing infant cognitive development. This study is significant because it is the first to examine breastfeeding, maternal depressive symptoms and alcohol use together, upon the association between maternal sensitivity and infant cognitive development, after adjusting for demographic covariates and infant birthweight. Maternal sensitivity, a measurable quality, advances infants’ cognitive development. Moreover, sensitivity and breastfeeding had independent effects upon cognitive development after controlling for multiple fixed and modifiable covariates. Understanding factors impacting the association between sensitivity and infant cognitive development provide avenues for developing more effective parenting interventions.

**Supplementary Information:**

The online version contains supplementary material available at 10.1186/s12887-022-03133-4.

## Background

The complex set of modifiable influences upon infant cognitive development remain important areas of study worldwide [1]. One universal factor, maternal sensitivity, also referred to as responsive parenting, is defined as a mother’s ability to observe her infant and respond appropriately to the “physical, emotional and developmental needs” of her child [2]. Maternal sensitivity is a measurable quality [2]. There is general consensus that maternal sensitivity encompasses at least four dimensions [3]: responds promptly and appropriately to the infant’s cues or signals [4]; alleviates the child’s distress [5]; demonstrates warmth [6]; and engages in developmentally appropriate play [7]. Research has repeatedly shown maternal sensitivity to be central to the development of infant cognitive development, or the ability of the infant to achieve developmental milestones such as babbling, smiling socially, and playing peek-a-boo [8]. Studies conducted in the United States have shown increased maternal sensitivity in the first year of life is associated with higher cognitive abilities [9], such as earlier achievement of language milestones [10], greater language comprehension [11], and increased infants’ persistence and problem-solving [12, 13]. Empirical support for the importance of maternal sensitivity in infancy is also demonstrated by enhanced primary school performance [14]; and decreased high risk youth behavior [15].

Maternal sensitivity has also been associated with breastfeeding [16, 17]. In one study that used Magnetic Resonance Imaging (MRI) to examine maternal brain activation in response to infant’s own cry, breastfeeding mothers showed greater activations in the superior frontal gyrus, insula, precuneus, striatum, and amygdala while listening to their own infant’s cries of distress as compared to formula-feeding mothers. However, there is uncertainty on whether highly sensitive mothers breastfeed, or if breastfeeding mothers are more likely to be responsive towards their infants [18]. This argument is supported by studies demonstrating that breastfeeding is associated with higher maternal education and income [19], factors also associated with sensitive parenting [20, 21]. Studies that examined the association between sensitivity and infant cognitive development without considering breastfeeding have been judged to overestimate the association between sensitivity and cognitive development [21].

Evidence from large randomized control trials examining the effectiveness of breastfeeding interventions also provide support that breastfeeding advances infant cognitive development [22]. Thus, we have ample research showing that in addition to maternal sensitivity, breastfeeding is a modifiable influence positively associated with infant cognition [23]. However, previous research has not been clear on the contribution of breastfeeding to advancing infant cognitive development given the association between maternal sensitivity and infant cognitive development [19, 21–23].

While breastfeeding is associated with both increased sensitivity and as well as advanced cognitive development, post-partum depression has been identified as a risk factor for both reduced sensitivity [24] and delayed infant cognitive development [25]. Paulson et al. (2010) [26] found that the number of depressive symptoms that mothers reported after the birth of their infant was associated with their increased negative affect, and a reduced ability to show warmth as well as less developmentally appropriate play.

Additionally, both maternal depression and breastfeeding are associated with maternal alcohol use [27]. Mothers who report depressive symptoms also report increased postpartum alcohol intake [27], while mothers who report breastfeeding also report lower alcohol consumption [28]. No studies have examined how maternal alcohol use affects the association between maternal sensitivity and infant cognitive development.

Studies that have examined the influence of breastfeeding or depression upon the association between sensitivity and infant cognitive development have typically used small or convenience samples. They have not had adequate sample sizes to adjust for multiple maternal demographic covariates as well as other factors associated with infant cognitive development, such as infant birth weight [29]..

This study, with a large population-based and nationally representative sample, examines multiple factors that modify infant cognitive development including maternal sensitivity, breastfeeding, and maternal depressive symptoms, while accounting for demographic covariates and infant birth weight. Findings inform how much breastfeeding and other factors contribute to the association between maternal sensitivity and cognitive development in the infant’s first year of life, and will aid in providing intervention recommendations across various parent subgroups.

## Methods

### Participants

**Fig. 1:**
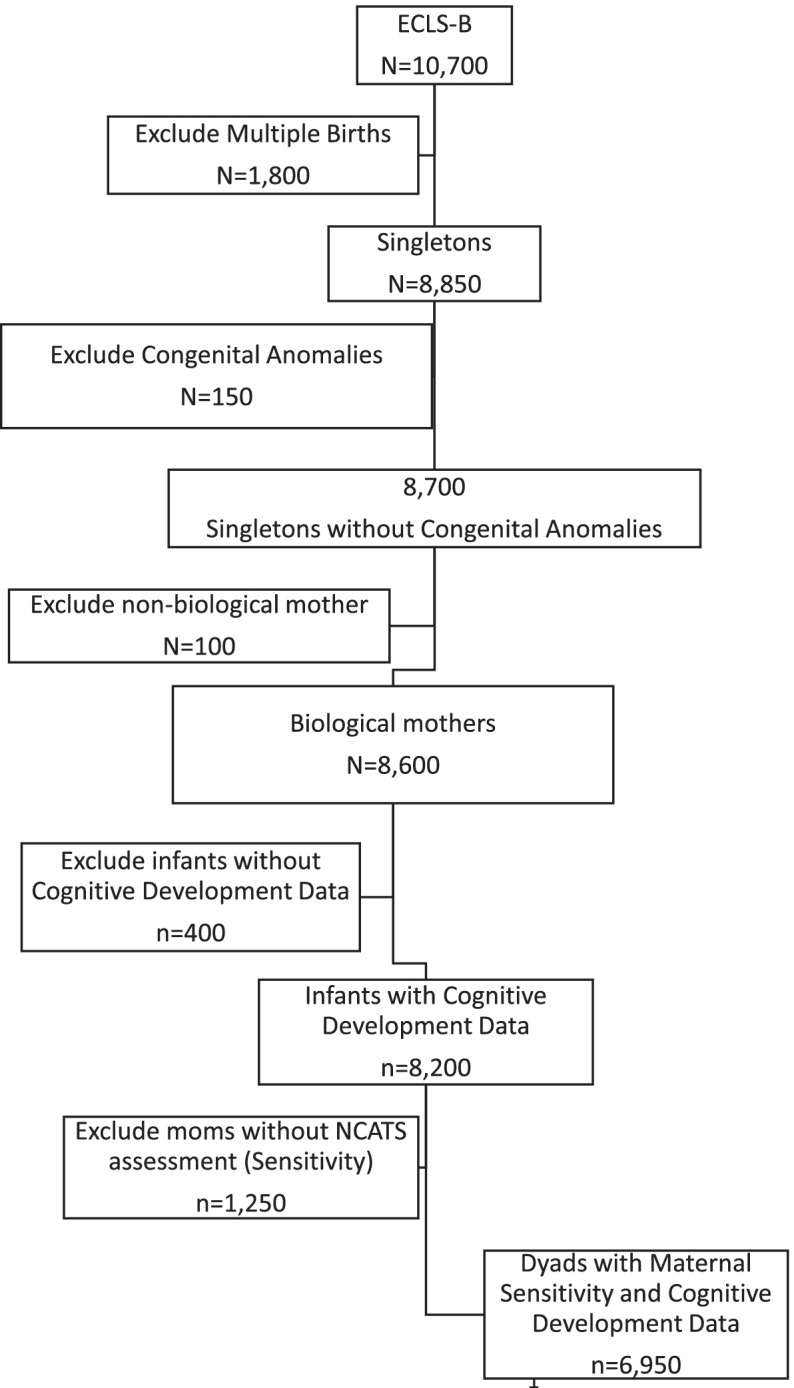
Analytic Sample. *rounded to nearest 50

This study used data collected during the first wave of the Early Childhood Longitudinal Study-Birth Cohort (ECLS-B) conducted by the National Center of Educational Statistics (NCES); infants were 9 months of age. Designed as a weighted nationally representative prospective study of factors that influence children’s development from birth to kindergarten, the base sample was selected using a 2-stage “clustered list frame approach”, and drawn from the approximately 4 million infants born in the USA in 2001 [30]. This sampling strategy was designed to oversample certain demographic groups (e.g., children born low or very low birth weight) and to first identify infants using birth certificates and then to define sampling units geographically over counties. A total of 76% (10, 668) of parents were interviewed of the 14,000 infant births between January and December 2001 that were initially sampled (Fig. 1). Roughly equal amounts of infant boys (51%) and girls (49%) have parent interviews after excluding infants with mothers less than 15 years of age, and infants who died or were adopted after birth. NCES provides sampling weights to correct for the sampling overestimates and the unequal probability of a child being selected for the study, and when sample weights are used, the ECLS-B data is representative of the US population of infants living with their biologic mothers age 15 or older in 2001 [31].

The final sample of this study included 6950 mother-infant dyads, where infants were singletons without congenital anomalies and their primary caregiver was the biological mother. Only dyads with complete data on both maternal sensitivity and infant cognitive development were included. Mothers excluded from the study (*n* = 1250) were more likely to have lower incomes (*p* = .02), be Asian or Hispanic (*p* = .00) and be single (*p* = .02) than mothers included in the study.

#### Procedures

Data was collected when infants were approximately 9 months old during home visits by trained researchers who used computer-assisted interview techniques. Researchers directly assessed infant development and videotaped mother-infant dyads while undertaking a semi-structured teaching task using the Nursing Child Assessment Teaching Scale (NCATS) protocol [32]. The NCATS protocol required mothers selecting one task from a list of items representing something the child did not know how to do yet, and to “teach” this task to their child. Mothers were encouraged to teach their infant for at least 45 s and to inform the trained researcher who was videotaping her when she was done with the teaching task. Videotaped mother-infant interactions were later coded by blinded coders.

### Exposure and outcome variables

*Maternal sensitivity* was assessed from videotaped observations of the mother-infant interaction using the (NCATS) Nursing Child Assessment Teaching Scale [32]. The NCATS measured maternal behaviors from 50 binary items (observed/not observed) grouped into four dimensions: Sensitivity to Cues (Ex: Mother positions child so that the child is safely supported); Response to Child’s Distress (Mother makes positive, sympathetic or soothing vocalization); Cognitive Growth Fostering (Ex: Mother uses at least 2 different sentences or phrases to describe the task to the child); and Socioemotional Growth Fostering (Ex: Mother laughs or smiles at child during the teaching interaction). NCATS scores were used as a continuous measure of maternal sensitivity. One item on the NCATS score rates the duration of the interaction (Mother spends no more than 5 min and not less than 1 min in teaching the child the task), and there is a small positive correlation between mothers’ sensitivity score and the time in which they engaged in the task (*r* = 0.14, *p* < .001) (*M* duration = 190.3 s, SD = 97.3).

NCATS coders did not conduct or attend home visits. All NCATS coders were blind to other measures collected on the dyads during the home visits. NCATS coders were trained and certified to code by the developers of the NCATS scale, University of Washington staff. NCATS ratings were also checked for quality by University of Washington staff, the developers of the scale. Coders were required to obtain 85% agreement or greater to continue scoring. Adequate internal consistency for the NCAST total scale was demonstrated for this sample (Cohen’s ∞ = .72) and full ECLS-B sample (Cohen’s ∞ = .68; NCES, 2005a).

*Infant cognitive development* was collected through direct child assessments by researchers, typically two, conducting the home visits using the Bayley Short Form – Research Edition (BSF-R), specifically designed for the ECLS-B using the Bayley Scales of Infant Development, Second Edition (BSID-II); a standardized assessment of developmental status for children from birth to 42 months of age.

NCES worked with the developers of the Bayley Scales of Infant Development to create the BSF-R using Item Response Theory to design the 31-item mental scale. The BSF-R included core items that all children were administered, and basal and ceiling items administered depending upon the child’s responses on core items. The mental scale assesses early cognitive and language ability through items on memory, communication and problem solving. Raw data responses on the BSF-R scale were used in the statistical analyses for this study. In the ECLS-B data set, BSF-R scale responses are equated to the full BSID-II mental scale (178 items) using Item Response Theory (IRT) and represent the number of items a child would have answered correctly if administered the full BSID-II mental scale [30]. The reliability of the BSF-R scale scores were high for this study sample (Cohen’s ∞ = .82) and the full ECLS-B sample (Cohen’s ∞ = 0.80) [33].

### Maternal factors

*Depressive symptoms* were measured using an abbreviated form of the Center for Epidemiological Studies Depression (CES-D) Scale [34]. This 12-item scale was the primary measure of mental health available in the ECLS-B for this study and has been validated in previous ECLS-B studies. Internal consistency for the CES-D is high (*α* = 0.82). This self-report scale assesses depressive symptoms during the past week using a four-point Likert scale: 0 = rarely/never, 1 = some/a little, 2 = occasionally/moderately, and 3 = most/all [25], yielding a total score ranging from 0 to 36. Sample questions include, “How many days in the past week have you …. had a poor appetite, felt depressed, felt lonely.” In research studies the CES-D is correlated with diagnosis of depression [34]. Higher scores correspond to greater depression; scores from 4 to 9 correspond to mild depression, and scores of 10 or higher correspond with moderate to severe depression. Examination the distribution of CES-D total score showed that data was distributed normally and therefore used as a continuous variable.

*Breastfeeding* items assessed by NCES for the ECLS-B (Additional file 1: Appendix A) asked if mothers ever breastfed their infant, if they were breastfeeding their child currently, and how many months they breastfed their infant (NCES, 2005). The raw data showed a high positively skewed distribution, with kurtosis =2.38. The raw distribution of the data justified creating a dichotomous variable: (1) not breastfed or breastfed less than a month or (2) breastfeeding 1 month or longer.

*Maternal Alcohol Use* data was collected as part of the parent interview in the ECLS-B and calculated as a categorical variable. Questions asked about mothers’ current consumption of alcohol: how often they drank, how many drinks they consumed per week, and how many drinks they had in one sitting in the past month (Additional file 1: Appendix B). Mothers were grouped into the following alcohol use categories: (1) Not currently drinking; (2) currently drinking; (3) currently drinking and had 1 or more times in the past month in which 4 drinks were consumed in one sitting.

### Covariates

*Infant sex* and *birth weight* were coded from the birth certificate data that was included in the ECLS-B. Very low birth weight was defined as less than 1500 g; low birth weight was defined as being between 1500 to 2499 g, and normal birth weight was defined as being greater than or equal to 2500 g.

*Parity* data was collected as part of the ECLS-B parent interview and was categorized into three groups: (1) target child only; (2) 2 to 3 children, including target child; (3) 4 or more children, including target child.

*Household income* and *poverty threshold* was obtained from parent interview. A continuous variable of income status created by the ECLS-B was used in analyses.

*Maternal age* data was collected as part of the parent interview. A continuous variable of maternal age was used in analyses.

*Race/Ethnicity of mother* data was extracted from the birth certificate and recoded into 5 categories in the ECLS-B: (1) non-Hispanic White, (2) non-Hispanic Black, (3) Hispanic, (4) Asian, and (5) Other.

*Maternal education* information was collected as part of the parent interview and categorized in the ECLS-B data set into 5 levels: (1) Less than high school, (2) High school diploma/(GED), (3) Vocational/trade school/some college, (4) College graduate, and (5) post-graduate.

*Family structure* data was collected as part of the parent interview. Responses were categorized into 2 categories: (1) Mother living with a male partner/father and (2) Mother living alone. Less than 100 mothers lived with the biological father in this sample.

### Data analyses

Statistical Analyses were conducted using SAS Version 9.4 (SAS, 2004).

Data estimates in the ECLS-B non-random sample may not be counted equally because not all mother-infant dyads had equal probability of selection. Sample and replicate weights provided by the ECLS-B and NCES were used to account for the sampling scheme, which over-represented certain demographic groups.

Descriptive analyses and correlational analyses (Table 1) were used to examine associations among demographic covariates, birth weight, depressive symptoms, breastfeeding, maternal alcohol use, maternal sensitivity, and infant cognitive development*.*

**Table 1:**
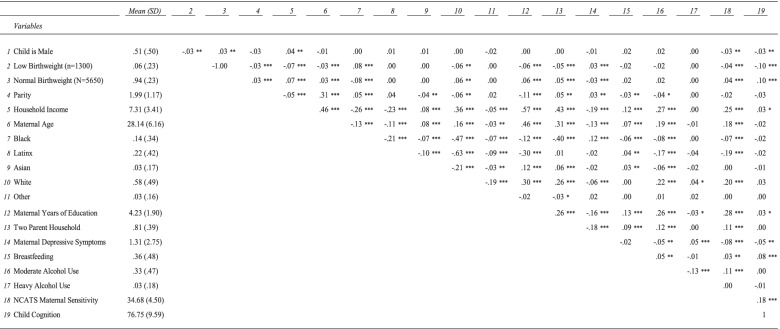
Correlations Between Study Variables

*p < .05; ** *p* < .01; ***p < .001; ~*p* < .10

Next, two sets of regression analyses were conducted. In the first set of regressions, maternal sensitivity was designated as a continuous outcome variable, and maternal depressive symptoms, breastfeeding, maternal alcohol use, infant birth weight and demographic covariates significantly associated with sensitivity in the correlational analysis, were entered as independent variables. In the second set of regression analyses, infant cognitive development was designated as a continuous outcome variable, and sensitivity, maternal depressive symptoms and breastfeeding and demographic covariates significantly associated in the correlational analysis, were entered as independent variables. All regressions were weighted with both the sample (W1CO) and the replicate weights (W1C1-W1C90). The jackknife method for estimating standard errors using replicate weights was also specified [35].

## Results

NCATS scores representing maternal sensitivity ranged from 15 to 49 (M = 34.36, SD = 4.54); BSF-R scores representing infant cognitive development ranged from 32.04 to 131.2 (M = 75.54; SD = 9.81), and CES-D scores representing maternal depressive symptoms ranged from 0 to 24 (M = 5.31; SD = 5.67). On the CES-D, over 90% of mothers had a score of 4 or below (indicating very few depressive symptoms), and over 70% of the sample had a score of either 0 or 1.

Correlation analysis (Table 1) showed infant’s sex (girls), birth weight, parity, household income, maternal race/ethnicity, maternal age, maternal education and family structure were significantly associated with higher maternal sensitivity scores. The absence of maternal depressive symptoms, breastfeeding for more than 1 month, and modest maternal alcohol use, were also significantly associated with greater maternal sensitivity scores.

Infant’s sex and birthweight, as well as household income, maternal age, the absence of maternal depressive symptoms, breastfeeding and maternal sensitivity were associated with higher infant cognitive development test scores.

Table 2 show the results of the weighted (sample and replicate weights) regression analyses conducted to determine the relationship of maternal and demographic covariates with the outcome of maternal sensitivity. Although depressive symptoms, breastfeeding and alcohol use were significantly associated with sensitivity in correlational analyses, after adjusting regression models for demographic covariates (infant sex [male], parity, household income, maternal race/ethnicity, age, education) and infant birth weight, these factors were no longer significantly associated with maternal sensitivity.

**Table 2:** Factors Associated with Maternal Sensitivity

***Standardized Beta*** ***Coefficient***	***β***	***β***	***β***	***β***	***β***	***β***	***β***	***β***	***β***	***β***	***β***	***β***	***β***	***β***	***β***
**Infant sex-male**	−.025*	−.026*	−.018	−.027*	−.025*	−.025*	−.022	−.021	−.021	−.019	−.019	−.019	−.019	−.019	−.019
**Birth Weight**		−.040**	−.038***	−.021	−.022	−.022	-..020	−.020	−.020	−.015	−.016	−.016	−.015	−.015	−.015
**Parity**			−.019	−.006	−.038**	−.037*	−.030*	−.032	−.032	−.002	−.002	−.002	−.001	−.001	−.001
**Household Income**				−.244***	−.197***	.195***	.159***	.159***	.159***	−.102***	.103***	.103***	.103**	.102**	.102**
**Age**					.095***	.095***	.089***	.091***	.091***	.038	.039	.039	.033	.033	.033
**Race** (White = Reference)															
**Black**						−.007	−.045***	−.048***	−.048***	−.048***	−.047***	−.046***	−.045***	−.045***	−.045***
**Hispanic**							−.137***	−.140***	−.140***	−.116***	−.115***	−.115***	−.116***	−.116***	−.116***
**Asian**								−.035**	−.035**	−.044**	−.043***	−.043**	−.042**	−.042**	−.042**
**Other**									−.003	−.001	−.001	−.001	.000	.000	.000
**Education (# of years)**										.159***	.159***	.159***	.158***	.158***	.158***
**Family Structure**															
Mother w Partner											.029	.029	.029	.029	.029
Mother no Partner												−.002	.002	.002	.002
**Depressive Symptoms** (No symptoms = Reference)															
Depressive Symptoms													−.019~	−.019~	−.019~
**Breastfeeding**	Less than 1 month = Reference														
Breastfeeding more than 1 month														.062	.062
**Alcohol Intake**															
Not drinking															
Drinks but4–5 drinks in one sitting															.015~
Has had 4–5 drinks 1 sitting															.043
**Model Summary (R**^**2**^**)**	.001***	.002***	.002***	.060***	.066***	.066***	.083***	.084***	.083***	.098***	.098***	.098***	.099***	.104***	.104***

*p < .05; ** *p* < .01; ***p < .001; ~*p* < .10

Regression models are weighted with both sample (W1CO) and replicate weights (W1C1-W1C90)

In a second set of weighted regressions conducted to examine the association between maternal sensitivity upon infant cognitive development, after controlling for demographic covariates (infant sex [male], infant birth weight, parity, household income, age, race/ethnicity education and family structure), maternal depressive symptoms, breastfeeding and alcohol intake, (Table 3), results showed maternal sensitivity (β = .175, *p* < .001) remained positively and significantly associated with infant cognitive development, even after breastfeeding (β = .079, *p* < .001) and depressive symptoms (β = −.035, *p* < .05), were included in the model (R^2^ = .053).

**Table 3:** Factors associated with Infant Cognitive Development

***Standardized Beta Coefficient***	***β***	***β***	***β***	***β***	***β***	***β***	***β***	***β***	***β***	***β***	***β***
**Infant sex-male**	−.033**	−.035**	−.036**	−.037**	−.037**	−.037**	−.037**	−.036**	−.031**	−.032**	−.033
**Birth Weight**		−.114***	−.112***	−.112***	−.111***	−.111***	−.111***	−.111***	−.107**	−.108**	−.108
**Poverty/Income**			.027*	.045**	.043**	.038**	.038**	.026	.007	.000	−.007
**Age**				−.039**	−.039**	−.039**	−.039**	−.046**	−.052***	−.057***	−.056
**Race** Reference = White											
**Black**					−.008	−.013	−.014	−.014	−.006	−.011	−.010
**Hispanic**						−.015	−.016	−.011	.011	.000	−.000
**Asian**							−011	−.013	−.004	−.008	−.009
**Other**								−.003	−.002	−.006	−.007
**Years of Education**								.029	−.002	−.003	−.012
**Sensitivity**											
									.180***	.175***	.175***
**Depressive Symptoms** Reference = No symptoms											
Depressive Symptoms										−.034**	−.035**
**Breastfeeding**	Reference = Less than 1 month										
Breastfeeding more than 1 month											.079**
**Model Summary (R**^**2**^**)**	.001***	.014***	.014***	.015***	.015***	.015***	.015***	.016***	.044***	.045***	.053***

*p < .05; ** *p* < .01; ***p < .001; ~*p* < .10

Regression models are weighted with both sample (W1CO) and replicate weights (W1C1-W1C90)

### Post-hoc analyses of maternal depressive symptoms and maternal alcohol use

Although there was little effect of depressive symptoms on the association between sensitivity and infant cognitive outcome, this finding may reflect the restricted range of depressive symptom scores in this sample. Less than 100 mothers reported symptoms outside of the mild depression range in this sample. Since the majority of mothers in this sample (over 70%) reported having either no depressive symptoms or only one depressive symptom, we were not able to evaluate the effects of moderate or severe depressive symptoms in this study.

Maternal alcohol use was not significantly associated with either maternal sensitivity or infant cognitive development. To determine whether the non-significant results of alcohol use were due to a lack of statistical power, a post hoc power analyses was conducted, with power (1 - β) set at 0.80 and α = 05, two-tailed. To have power to detect a mean difference in cognitive development scores of infants of mothers who reported drinking in comparison to those who did not, 6100 participants in each group would be required. However, the power in this study was 34%. Thus, though maternal alcohol use was not associated with either sensitivity or infant cognitive development, there may have been inadequate power in the sample to ensure the lack of significant association between alcohol use and either sensitivity or cognitive development.

## Discussion

This population-based study is positioned to untangle the effects of the multiple factors that influence infants’ cognitive development. Research conducted in high income countries demonstrates that high maternal sensitivity sets in motion a chain of events based in reciprocal interactions with the infant that lays a foundation for later school and occupational success. Breastfeeding has also been previously associated with more rapid cognitive development, while maternal depressive symptoms has been negatively associated with cognitive development. No previous study has examined the effect of maternal alcohol use, associated with both maternal depressive symptoms and breastfeeding with either maternal sensitivity or infant cognitive development [1].

The main finding showed that an independent, significant and positive association remained between maternal sensitivity and infant cognitive development, after adjusting for multiple covariates. Breastfeeding was not significantly associated with maternal sensitivity after adjusting for covariates in regression analyses. The relationship between sensitivity and infant cognitive development remained significant even after breastfeeding was added into the model, indicating the breastfeeding contributed unique variance to the outcome of infant cognitive development. Findings from this study challenge the notion the association between sensitivity and cognitive development is accounted for by breastfeeding [18], and suggest breastfeeding is an independent and separate means from sensitivity to advancing infant cognitive development.

Results did not indicate a significant association between maternal depressive symptoms with maternal sensitivity. This lack of association may be related to the low numbers of mothers in this sample who reported having depressive symptoms.

There was also no significant association between maternal alcohol use and infant cognitive development. However, most mothers in this sample did not report drinking more than 1 drink/week in the home. This may indicate biased reporting due to potential stigma, where mothers might systematically under report drinking in this study. Post-hoc power analyses suggested that there was not sufficient power to detect an effect between mean scores of mothers of infants who reported drinking with those who did not.

Documentation of the effects of maternal sensitivity and factors such as breastfeeding upon the development of both healthy and biologically vulnerable infants is key to mobilizing resources and developing and designing appropriate and effectively tailored interventions and policies to ultimately enrich child outcomes globally. This research may suggest that a brief screening tool for maternal sensitivity and breastfeeding practices would be helpful in a clinical setting that is supporting the advancement of infant cognitive development.

### Limitations

Mother-infant dyads were excluded from the sample if they were missing either maternal sensitivity or infant cognitive development data. Dyads excluded from the study were not significantly different from those included, however, they were more likely to have lower household income, be black, Hispanic or Asian, have a high school education or less, and be single mothers. The somewhat lower participation rate of these under resourced groups of mothers (low-income, non-white, single parent) may have introduced selection bias. Still, a substantially large number of single, low-income, black Hispanic and Asian groups, high school graduates remained in the study sample. In addition, these demographic factors were adjusted for in statistical analyses.

This study contained a rich array of measures. Although the CES-D measure of depressive symptoms has been validated in previous studies, it is a self-report measure of symptoms, and does not provide diagnostic information. We regret that we did not have additional measures of mental health. Two other constructs, breastfeeding and alcohol intake were also assessed using self-reported interview, and thus prone to response bias. Another limitation was that few questions assessed breastfeeding. Additionally, it is not clear if the breastfeeding was done exclusively or in conjunction with solid foods.

Lastly, although respondents were told that their responses would not be individually identifiable and would be reported in the aggregate, respondents may have believed they need to respond to the question in a socially desirable manner, have difficulty in understanding survey questions, or have problems with adequate recall.

### Study strengths

The ECLS-B is a population based nationally representative dataset which allowed us to study under-researched topics such as whether breastfeeding accounted for the association between maternal sensitivity and cognitive development and provides adequate sample size needed to control for multiple sociodemographic variables. The ECLS-B has the additional strength of having minimal missing data on demographic covariates.

Study measures are taken from valid and reliable instruments. Moreover, for both the maternal sensitivity measure (NCATS) and the infant cognitive developmental test (BSF-R), each administrator’s testing and scoring abilities were validated both through in-person quality control visits as well as reliability coding of videotaped interviews.

## Conclusion

Maternal sensitivity remained strongly and positively associated with infant cognitive development, even after controlling for breastfeeding, multiple demographic covariates and infant birth weight. Therefore, and importantly, although breastfeeding itself was significantly associated with cognitive development, it did not alter the strong and positive association between maternal sensitivity and cognitive development. Both sensitivity and breastfeeding are separate means to advancing infant cognition and should be emphasized in parenting interventions involving young infants.

### Future directions

More research is needed on effective maternal behavior, such as maternal sensitivity and its association with overall development, health and survival of infants, particularly in high-risk conditions, such as mothers with mental illness, substance abuse issues, or risk factors relating to the infant, such as low birth weight. The question of modifiable factors, such as moderate to severe maternal depression and alcohol use, remain unanswered in this study. Future directions should also include examining data longitudinally to investigate if sensitivity and breastfeeding continue to be associated with more rapid cognitive development at later ages.

## Supplementary Information

**Additional file 1.**

## Data Availability

The data that support the findings of this study are available from the National Center for Education Statistics’ (NCES’) Early Childhood Longitudinal Study - Birth Cohort (ECLS-B) (https://nces.ed.gov/ecls/birth.asp), but restrictions apply to the availability of these data, which were used under license for the current study, and so are not publicly available. Data are available only to qualified researchers granted an IES restricted-use data license upon request and with permission of NCES (https://nces.ed.gov/ecls/birthdatainformation.asp).

## Abbreviations

ECLS-B: Early Childhood Longitudinal Study-Birth Cohort
NCES: National Center for Educational Statistics
NCATS: Nursing Child Assessment Teaching Scale
BSF-R: Bayley Short Form-Research Edition
BSID-II: Bayley Scale of Infant Development, II
CES-D: Center for Epidemiological Studies’ Depression Scale
