# Supplementary material for: Does breastfeeding account for the association between maternal sensitivity and infant cognitive development in a large, nationally representative cohort?
Source: BMC Pediatr. 2022 Jan 26;22:61. doi: 10.1186/s12887-022-03133-4 (PMC8790903; doi:10.1186/s12887-022-03133-4)
Supplement: Supplementary file 1 — Additional file 1. [file 12887_2022_3133_MOESM1_ESM.docx]

**Appendix A**

**Breastfeeding Questions/Measures**

1. **Did you ever breast-feed CHILD?**
2. **Are you still breast-feeding CHILD now?**
3. **For how many months did you mother breast-feed (him/her)?**
4. **During the past 7 days, was CHILD breast-fed formula-fed, or fed regular cow’s milk?**
5. **How old was CHILD in months when you began feeding (him/her) formula?**
6. **How old was CHILD in months when you began feeding (him/her) cow’s milk?**
7. **How old was CHILD in months when solid food was first introduced? Solid foods include cereal and baby food in jars, but not finger foods.**
8. **How old was CHILD in months when (he/she) was first given finger foods, such as Cheerios, teething biscuits, crackers, bread, noodles, rice, grits, tortillas, or potatoes?**

**Appendix B**

**Alcohol Intake Questions/Measures**

1. **In the 3 months before you got pregnant, how many alcoholic drinks did you have in an average week?**

PROBE: A drink is: One glass of wine, one wine cooler, one can or bottle of beer, one shot of liquor, or

one mixed drink.

1. **During the last 3 months of your pregnancy, how many alcoholic drinks did you have in an average week?**
2. **Do you currently drink any alcoholic beverages?**
3. **How many alcoholic drinks do you have in an average week now?**
4. **In the last month, how many times did you drink (four/five) or more alcoholic drinks at one sitting?**

**Breast Feeding**
